# Both CD4^+^ and CD8^+^ Lymphocytes Participate in the IFN-*γ* Response to Filamentous Hemagglutinin from *Bordetella pertussis* in Infants, Children, and Adults

**DOI:** 10.1155/2012/795958

**Published:** 2012-04-08

**Authors:** Violette Dirix, Virginie Verscheure, Françoise Vermeulen, Iris De Schutter, Tessa Goetghebuer, Camille Locht, Françoise Mascart

**Affiliations:** ^1^Laboratory of Vaccinology and Mucosal Immunity, Université Libre de Bruxelles (ULB), 1070 Brussels, Belgium; ^2^Pediatric Department, Hôpital Erasme, Université Libre de Bruxelles (ULB), 1070 Brussels, Belgium; ^3^Pediatric Department, UZ Brussel, Vrije Universiteit Brussel (VUB), 1090 Brussels, Belgium; ^4^Pediatric Department, Hôpital Saint-Pierre, Université Libre de Bruxelles (ULB), 1000 Brussels, Belgium; ^5^INSERM U1019, 59000 Lille, France; ^6^CNRS UMR8204, 59021 Lille, France; ^7^Center for Infection and Immunity of Lille, Institut Pasteur de Lille, 59019 Lille, France; ^8^Univ Lille Nord de France, 59000 Lille, France; ^9^Immunobiology Clinic, Hôpital Erasme, Université Libre de Bruxelles (ULB), 1070 Brussels, Belgium

## Abstract

Infant CD4^+^ T-cell responses to bacterial infections or vaccines have been extensively studied, whereas studies on CD8^+^ T-cell responses focused mainly on viral and intracellular parasite infections. Here we investigated CD8^+^ T-cell responses upon *Bordetella pertussis* infection in infants, children, and adults and pertussis vaccination in infants. Filamentous hemagglutinin-specific IFN-**γ** secretion by circulating lymphocytes was blocked by anti-MHC-I or -MHC-II antibodies, suggesting that CD4^+^ and CD8^+^ T lymphocytes are involved in IFN-**γ** production. Flow cytometry analyses confirmed that both cell types synthesized antigen-specific IFN-**γ**, although CD4^+^ lymphocytes were the major source of this cytokine. IFN-**γ** synthesis by CD8^+^ cells was CD4^+^ T cell dependent, as evidenced by selective depletion experiments. Furthermore, IFN-**γ** synthesis by CD4^+^ cells was sometimes inhibited by CD8^+^ lymphocytes, suggesting the presence of CD8^+^ regulatory T cells. The role of this dual IFN-**γ** secretion by CD4^+^ and CD8^+^ T lymphocytes in pertussis remains to be investigated.

## 1. Introduction

The immune system of neonates and young infants differs in many aspects from that of adults, which has important implications for the development of effective and safe immune-interventions and vaccination in early life. Compared to that of adults, the neonatal adaptive immune system is immature with respect to both B- and T-cell responses [1–4], the latter being partially impaired as a result of a functional alteration of neonatal antigen presenting cells (APCs) [5]. Whereas several vaccines administrated during infancy require the development of antigen-specific IFN-*γ* responses to be protective, the CD4^+^ T-cell responses in newborns and infants appear to be most often biased towards the Th-2 type. Upon stimulation with the vaccine antigens, the CD4^+^ T-cell responses are often characterized by the production of IL-4, IL-5, and IL-13 at the expense of IFN-*γ* [6, 7].

However, in certain circumstances, even very young infants are able to mount a robust Th-1 type response, with high levels of IFN-*γ* secretion, and minimal amounts of Th-2 cytokine production. It has been demonstrated that vaccination at birth with *Mycobacterium bovis* Bacille Calmette-Guérin (BCG) induces INF-*γ* responses to purified protein derivative at levels similar to those induced by BCG vaccination in older children and in adults [8]. Natural infection with the whooping cough agent *Bordetella pertussis* also induces strong *B. pertussis*-antigen-specific IFN-*γ* responses in very young infants, with no detectable IL-13 or IL-4 production [9].

Studies on antigen-specific IFN-*γ* responses in infants have so far mostly focused on CD4^+^ T cells, and much less is known about the capacity of infants to induce CD8^+^-IFN-*γ*-producing cells. Compared to adults, infants have significantly reduced CD8^+^ T-lymphocyte proportions in their peripheral blood mononuclear cell (PBMC) population [3, 10]. Nevertheless, strong CD8^+^ T-cell responses have been detected in infants congenitally infected with cytomegalovirus [11] or *Trypanosoma cruzi* [12]. However, both infectious agents are primarily or exclusively intracellular pathogens, which is likely one of the reasons for their ability to induce strong CD8^+^ T-cell responses. Virtually nothing is known about the potential of extracellular pathogens or protein vaccines to induce CD8^+^ T-cell responses in infants or early childhood.

In this study, we used pertussis vaccination and disease to address this issue. Despite wide vaccination coverage with efficacious vaccines, pertussis or whooping cough cases still mount to up to 50 million per year, with nearly 300,000 annual deaths recorded worldwide [13]. We and others have previously reported a strong production of IFN-*γ* in response to *in vitro* stimulation with *B. pertussis* antigens, such as filamentous hemagglutinin (FHA) of PBMC from children presenting with whooping cough [9, 14–16] or vaccinated with pertussis vaccines [17–20], including preterm infants with very low gestational age [21]. Here, we have characterized the phenotype of the IFN-*γ*-producing lymphocytes in response to FHA in a cohort of acutely *B. pertussis*-infected infants and young adults, as well as in pertussis-vaccinated children, to determine whether CD8^+^ lymphocytes may play a significant role in *B. pertussis*-specific IFN-*γ* responses in infants, compared to older children and adults.

## 2. Material and Methods

### 2.1. Study Participants and Blood Collection

A total of 31 *B. pertussis*-infected subjects and 27 vaccinated children were enrolled in the study. Among the infected subjects, 25 were infants younger than 1 year of age (median, range in months: 2, 0–7), and 6 were adolescents or young adults (median, range in years: 12.5, 11–32). All cases were confirmed by polymerase-chain reaction (PCR) and/or *B. pertussis *culture, and blood was collected during the acute phase of the disease. Among the 27 vaccinated children, 4 had received the cellular vaccine Tetracoq (sanofi pasteur, Lyon, France), 17 had received the acellular vaccine Tetravac (sanofi pasteur, France), and 6 had received the acellular vaccine Infanrix hexa (GSK Biologicals, Rixensart, Belgium). Some of the vaccinated children had already been included in the analysis of other parameters of the immune response to pertussis vaccines [22, 23]. All infants followed the vaccination schedule according to the Belgian recommendation, and blood samples were collected at a median age of 11 months (range: 3–18 months). Therefore, most of them had received at least 3 vaccine injections, except two infants tested after only 2 vaccine injections, and two others tested after having received the first booster dose. All vaccinated children were HIV-negative but were *in utero* HIV-exposed and therefore received a preventive therapy with zidovudine during the first 6 weeks of life.

This study was approved by the ethical committees from the Hôpital Saint-Pierre (Brussels, Belgium) and the Hôpital Erasme (Brussels, Belgium), and informed parental consent was obtained. Due to the small volume of blood, not all analyses could be performed for all the subjects.

### 2.2. Antigens, Mitogen, and Blocking Antibodies for Cellular Immune Assays

FHA was purified by heparin-Sepharose chromatography as described [24] from pertussis toxin-deficient *B. pertussis* BPRA [25], and its concentration was estimated by BCA Protein Assay Reagent Kit (Pierce Biotechnology, Rockford, USA). FHA was used at 1 or 5 *μ*g/mL, as specified for the *in vitro* stimulation. Phytohemagglutinin (PHA) (Remel, Lenexa, KS, USA) was used as a positive control at 2 *μ*g/mL.

When indicated, blocking anti-human MHC-I or -II antibodies (mouse anti-human HLA-ABC antigen, clone W6/32 or mouse anti-human HLA-DP, DQ, DR antigen, clone CR3/43, respectively, DAKO Diagnostics S.A., Heverlee, Belgium) or the corresponding isotype control antibodies (mouse IgG1 or IgG2a, DAKO Diagnostic S.A., Belgium) were added in the culture medium at 2 *μ*g/mL.

### 2.3. Cell Isolation and Culture: Cytokine Concentration Determination

Blood samples were processed within 4 h following the puncture. PBMC were isolated from whole blood by density gradient centrifugation on lymphoprep (Nycomed Pharma, Oslo, Norway) and cultured at 2 × 10^6^/mL with the stimulating antigen/mitogen in supplemented RPMI medium at 37°C under 5% CO_2_, as previously described [9]. After 72 h, supernatants were collected, and IFN-*γ* production was measured using sandwich enzyme-linked immunosorbent assays (ELISAs), according to the manufacturers' instructions (Kit IFN-*γ* cytoset ELISA, BioSource International, Camarillo, CA, USA). IFN-*γ* concentrations were calculated using the KC4 software (BRS, Drogenbos, Belgium) by referring to a standard curve generated by serial dilutions of the standard, from 2,500 pg/mL to 10 pg/mL IFN-*γ*. When detectable, IFN-*γ* concentrations obtained under nonstimulated conditions were subtracted from those obtained for mitogen- or antigen-stimulated cells.

### 2.4. Lymphocyte Depletions

CD4^+^ or CD8^+^ T lymphocytes were depleted by incubating whole blood with anti-CD4 or anti-CD8 antibodies for 20 minutes at room temperature (RosetteSep, StemCell Technologies, Grenoble, France), followed by a density gradient centrifugation on lymphoprep (Nycomed Pharma, Norway). The effectiveness of the depletion was controlled by flow cytometry (FACSCalibur or FACSCanto, BD Biosciences, Mountain view, CA, USA) after staining the cells with a combination of anti-human monoclonal antibodies (anti-CD3-PerCp-Cy5.5, anti-CD4-APC, anti-CD8-FITC, anti-CD14-PE, BD Biosciences, USA). Percentages of remaining CD4^+^ T cells in the CD4^+^-depleted cell suspensions were always lower than 3% of the total T lymphocytes, and percentages of the remaining CD8^+^ lymphocytes were always lower than 0.7% in the CD8^+^-depleted cell suspensions. The median of CD14^+^ percentages after CD4^+^ T-cell depletion was 10.3% (range = 5.3–28%) of the whole PBMC. The analyses were performed using cellquest (BD Biosciences, USA) or FlowJo (Tree Star, Ashland, OR, USA).

### 2.5. Phenotyping of FHA-Specific IFN-*γ*-Containing Cells

Isolated PBMC were stimulated overnight with 5 *μ*g/mL of FHA in the presence of 2 *μ*g/mL of each of the costimulatory antibodies *α*-CD28 (clone L293, BD Biosciences, USA) and *α*-CD49d (clone L25.3, BD Biosciences, USA). 10 *μ*g/mL Brefeldin-A (Sigma Aldrich, Saint Louis, MO, USA) was added for the last 4 h of incubation. After fixation and permeabilization of the cells according to the manufacturers' instructions (Lysing-Solution1 and Permeabilizing-Solution2, BD Biosciences, USA), PBMC were stained with anti-human monoclonal antibodies: anti-CD3-PerCp-Cy5.5, anti-CD4-APC-Cy7, anti-CD8-APC, and anti-IFN-*γ*-PE (BD Biosciences, USA). Stained cells were examined using a FACSCanto flow cytometer (BD Biosciences, USA), and the data were analyzed by using the FlowJo software (Tree Star, USA). When detectable, percentages of IFN-*γ*-containing CD4^+^ or CD8^+^ CD3^+^ T lymphocytes obtained under nonstimulated conditions (median = 0.1%) were subtracted from those obtained for FHA-stimulated cells. Both percentages and absolute numbers of CD4^+^- or CD8^+^-containing IFN-*γ* were evaluated. Absolute numbers of positive cells reported for 1,000,000 CD3^+^ T cells were calculated as follows:

(1) $$\begin{array}{cc} & \left(\text{\%}\;\text{FHA-induced}\;\text{IFN-}\gamma \;\text{producing}\;\text{CD}4^{+}\;\text{or}\;\text{CD}8^{+}\;\text{cells}\right)\\ & \;\;\;\times \left(\text{\%}\;\text{CD}4^{+}\;\text{or}\;\text{CD}8^{+}\;\text{cells}\;\text{within}\;\text{CD}3^{+}\;\text{T}\;\text{cells}\right)\\ & \;\;\;\times \frac{10^{6}}{100}. \end{array}\;\;$$

### 2.6. Statistical Analysis

The statistical analyses were performed using the GraphPad Prism version 4.00 for Windows (GraphPad Software, San Diego, CA, USA, http://www.graphpad.com/). The significance of differences between different series of results was determined using nonparametric Wilcoxon matched pairs test.

## 3. Results

### 3.1. Effect of Blocking Anti-MHC Class-I and Class-II Antibodies on the IFN-*γ* Production Induced by FHA

**Figure 1:**
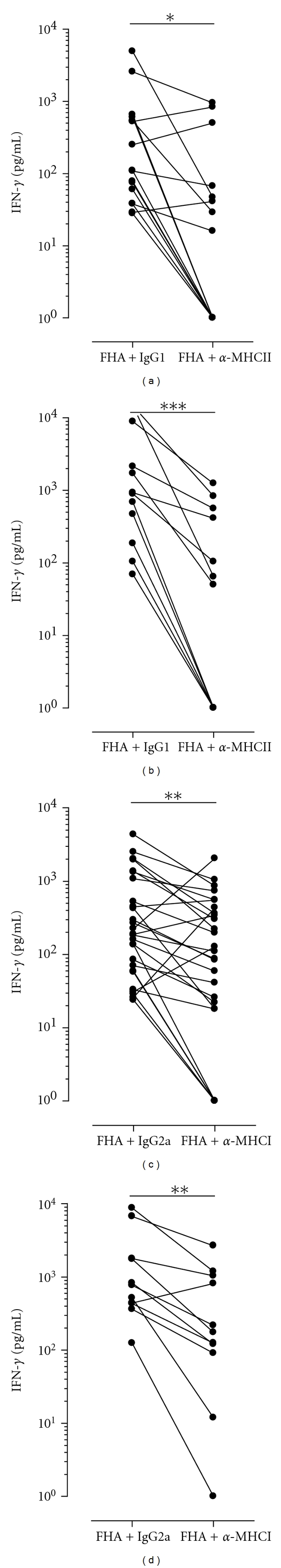
Effect of blocking anti-MHC class I and anti-MHC class II antibodies on the FHA-specific IFN-*γ* production. Isolated PBMC were stimulated *in vitro* for 72 h with 1 *μ*g/mL FHA in the presence or absence of blocking anti-MHC-II (a) and (b) or anti-MHC-I (c) and (d) antibodies or in the presence or absence of control isotypes. Unstimulated cells were tested as control. IFN-*γ* concentrations were measured in the cell culture supernatants and values obtained for unstimulated cells were subtracted from those obtained for stimulated cells. Circles represent the individual IFN-*γ* concentrations obtained after stimulation with FHA in the presence of anti-MHC antibodies or control isotypes. Results obtained for the same samples are linked. *P < 0.05; **P < 0.001; ***P < 0.0001.

As a first approach to analyse the possible contribution of CD4^+^ and/or CD8^+^ T lymphocytes in the FHA-induced IFN-*γ* production, we analysed the MHC restriction of this IFN-*γ* secretion by testing the effect of blocking antibodies against the class I and class II MHC molecules. The FHA-induced IFN-*γ* concentrations obtained in the presence of these blocking antibodies were compared to those obtained in the presence of isotype controls (Figure 1). Compared to the addition of an isotype control to the FHA-stimulated PBMC, the addition of an anti-MHC-II antibody inhibited the IFN-*γ* secretion for both *B. pertussis-*infected subjects (*P* = 0.0186, Figure 1(a)) and vaccinated children (*P* = 0.0005, Figure 1(b)). A total inhibition of the FHA-induced IFN-*γ* secretion in the presence of anti-MHC-II antibodies was noted for 8/15 infected patients and for 5/12 vaccinated children. In contrast, no inhibition was observed for 3 infected children but their FHA-induced IFN-*γ* secretion was inhibited by the addition of an anti-MHC-I antibody suggesting that in these cases, CD8^+^ lymphocytes were the predominant IFN-*γ* producing lymphocyte subset. The results obtained in the presence of anti-MHC-II antibodies were similar for infected infants, children, and adults as well as for children vaccinated with an acellular vaccine and with a whole cell vaccine (data not shown). 

Anti-MHC-I antibodies also inhibited the FHA-induced IFN-*γ* secretion compared to the addition of an isotype control, both in infected children (*P* = 0.0079, Figure 1(c)) and in vaccinated children (*P* = 0.0034, Figure 1(d)). The inhibition was total for some children and partial for others with no difference according to the children's age. Interestingly, for 6 children (5 infected and 1 vaccinated), the FHA-induced IFN-*γ* concentrations were noticeably higher in the presence of an anti-MHC-I antibody than in the presence of the isotype control (Figures 1(c) and 1(d)).

These results indicate that both the MHC class-I and MHC class-II molecules are involved in the recognition of FHA, and that they are both required for the optimal secretion of IFN-*γ* by PBMC in response to stimulation with FHA. This suggests the involvement of both CD8^+^ and CD4^+^ lymphocytes in the FHA-induced IFN-*γ* secretion.

### 3.2. Phenotyping of FHA-Specific IFN-*γ*-Producing Cells

To confirm that both CD4^+^ and CD8^+^ lymphocytes participate in the IFN-*γ* synthesis induced by FHA, the phenotype of the cells was analysed by flow cytometry after simultaneous immunostaining of intracellular IFN-*γ* and surface markers on FHA-stimulated cells for 10 children (6 infected and 4 vaccinated).

**Figure 2:**
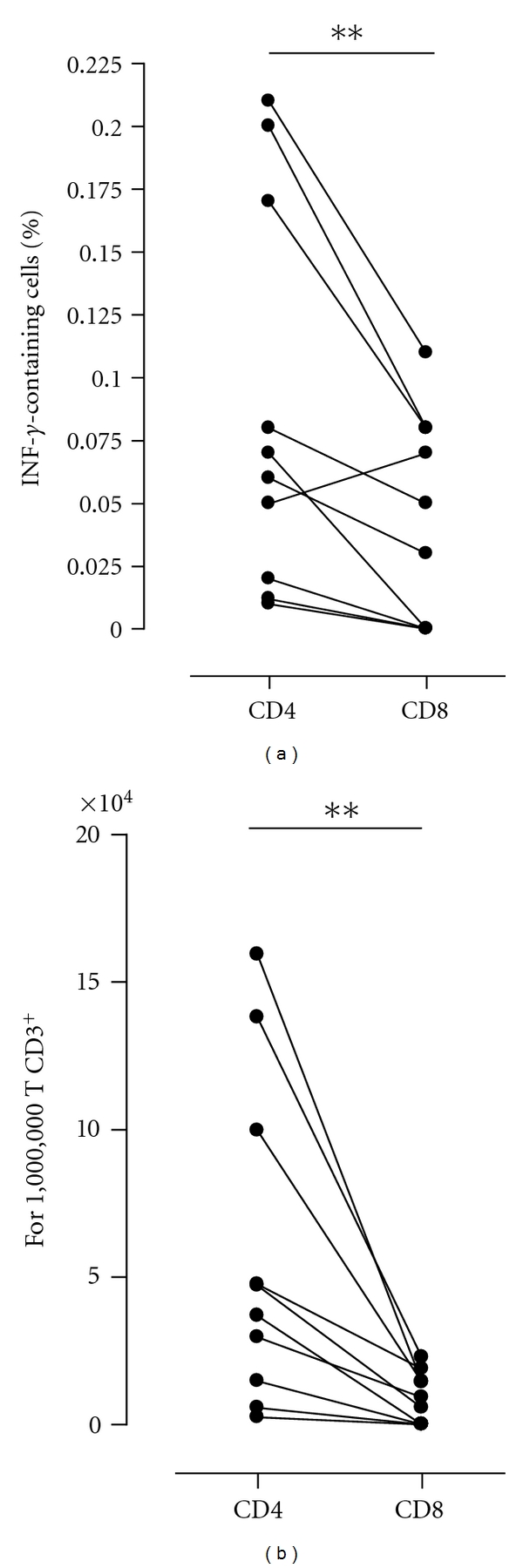
Percentages and absolute numbers of FHA-induced IFN-*γ* producing CD4^+^ and CD8^+^ T cells in *B. pertussis*-infected (*n* = 6) or vaccinated children (*n* = 4). Isolated PBMC were stimulated *in vitro* for 24 h with 5 *μ*g/mL FHA, and IFN-*γ*-containing CD4^+^ (left columns) and CD8^+^ (right columns) T lymphocytes were detected by flow cytometry. (a) represents the percentages of positive cells and the absolute numbers of IFN-*γ*-producing cells expressed for 10^6^ CD3^+^ T lymphocytes are shown on (b). The lines join the results obtained for the same children. Percentages obtained for unstimulated cells were subtracted from those obtained for stimulated cells. *P < 0.05, **P < 0.001.

The percentages and absolute numbers of CD4^+^ and CD8^+^-IFN-*γ* containing lymphocytes obtained in response to a stimulation with FHA and after subtraction of the values obtained for nonstimulated cells are represented on Figure 2. Whereas IFN-*γ*-containing CD4^+^ lymphocytes were detected for all the subjects (median and 25th–75th percentiles of the IFN-*γ*-containing-CD3^+^CD4^+^ cells: 0.06% and 0.01–0.21%, resp.), IFN-*γ*-containing CD8^+^ lymphocytes were only detected for 6/10 children (3/6 infected and 3/4 vaccinated) (median and 25th–75th percentiles of the IFN-*γ*-containing-CD3^+^CD8^+^ cells: 0.04% and 0.00–0.11%, resp.), resulting therefore in a significantly lower percentage of IFN-*γ*-containing CD8^+^ compared to CD4^+^ lymphocytes (*P* = 0.0164) (Figure 2(a)). 

**Figure 3:**
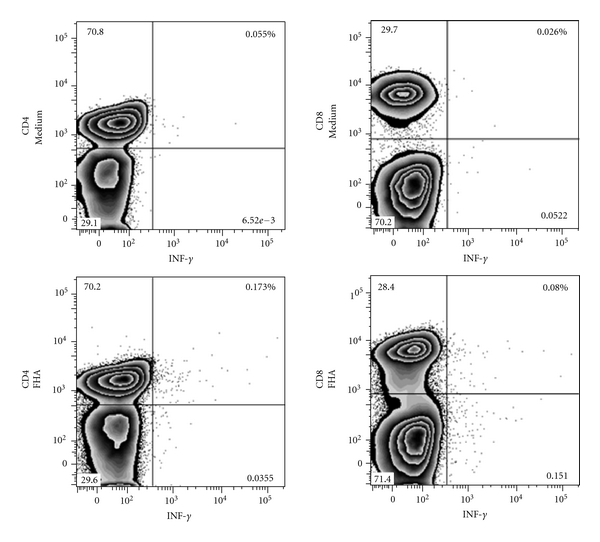
Dot-plot of the flow cytometric analyses of the phenotype of FHA-induced IFN-*γ* producing cells for one representative subject. Isolated PBMC were cultured *in vitro* for 24 h in the presence (lower panels) or absence (upper panels) of FHA. CD4^+^ T cells (left panels) or CD8^+^ T cells (right panels) were gated in the CD3^+^ T lymphocytes. Results are expressed as the percentages of IFN-*γ* producing cells among the cell subpopulation analyzed.

 As the numbers of circulating CD4^+^ lymphocytes are normally higher than the numbers of CD8^+^ lymphocytes, even more significant differences between the contributions of CD4^+^ and CD8^+^ lymphocytes to the FHA-induced IFN-*γ* synthesis were noted when comparing the absolute numbers of IFN-*γ*-containing-T lymphocytes (*P* = 0.0020, Figure 2(b)). Although it has not been possible to analyze the amounts of IFN-*γ* produced by individual cells, taken together with the data shown in Figure 1, these results indicate that the CD4^+^ T cells are the major source of FHA-specific IFN-*γ*, yet for some children the CD8^+^ cells contribute significantly to the IFN-*γ* production. A representative dot plot of the unstimulated versus FHA-induced IFN-*γ* synthesis in CD4^+^ and CD8^+^ lymphocytes is shown on Figure 3.

### 3.3. CD4^+^-CD8^+^ T-Lymphocyte Interactions in the FHA-Induced IFN-*γ* Secretion

**Figure 4:**
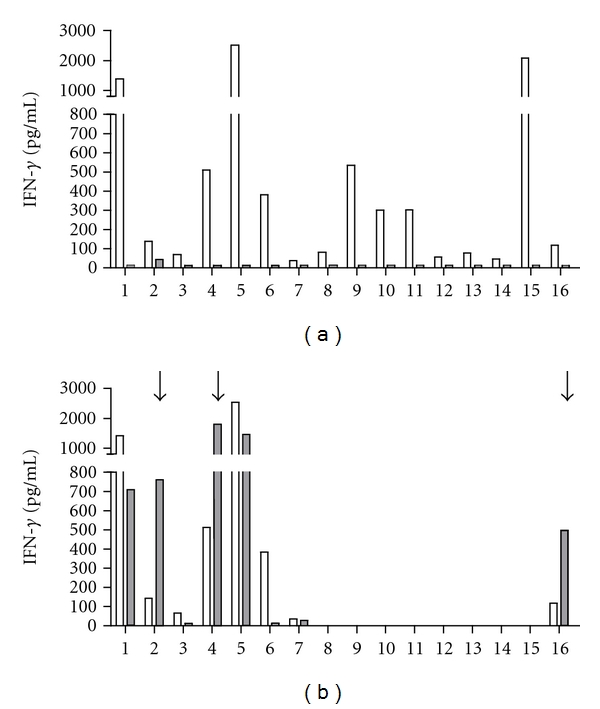
Effect of the CD4^+^ or CD8^+^ depletion on the FHA-induced IFN-*γ* production by PBMC from *B. pertussis*-infected or vaccinated children. Isolated PBMC were stimulated *in vitro* for 72 h with 1 *μ*g/mL FHA after CD4^+^ (a) or CD8^+^ (b) depletion. Results from infected children are represented by columns 1 to 5 whereas those from vaccinated children are indicated by numbers 6 to 16. Open columns represent the values obtained for the nondepleted cell suspension and grey columns those obtained after CD4^+^ or CD8^+^ depletions. The arrows indicate the samples characterized by a higher IFN-*γ* secretion in CD8-depleted cell suspension compared to the PBMC.

The CD4^+^ T cells or the CD8^+^ T cells were depleted from the PBMC of 5 infected infants before *in vitro* stimulation with FHA. We also depleted the CD4^+^ T cells from 11 vaccinated children, and the CD8^+^ T cells from 3 of them before *in vitro* stimulation with FHA. The PBMC and the CD4^+^-depleted and the CD8^+^-depleted cell suspensions were then cultured in the presence or absence of FHA for 72 h, and the IFN-*γ* concentrations present in the cell culture supernatants were measured by ELISA. The effect of the depletions was assessed by comparing the IFN-*γ* concentrations in the depleted cell culture to those of the whole PBMC supernatants. As shown in Figure 4, depletion of the CD4^+^ T cells resulted in a total loss of the FHA-specific IFN-*γ* production for all but one child (n°2), suggesting that the IFN-*γ* secretion by CD8^+^ lymphocytes requires the help from CD4^+^ lymphocytes. The FHA-induced IFN-*γ* concentrations were therefore significantly lower for the CD4^+^-depleted cell suspensions compared to the PBMC (*P* = 0.0005). The residual FHA-induced IFN-*γ* secretion by the CD4^+^-depleted cell suspension for one subject (n°2, 12-year-old-infected child) could perhaps be attributed to IFN-*γ* secretion by double negative cells, such as NK cells or by *γδ* T lymphocytes, that were still present in the CD4^+^-depleted cell suspension. However, due to limited amounts of cells available, this could not be further investigated.

In contrast, results obtained after the depletion of the CD8^+^ T cells were highly variable with an absence of residual IFN-*γ* secretion for 2/8 subjects (1 infected and 1 vaccinated), lower but detectable levels of IFN-*γ* secretion compared to the PBMC for 3/8, and higher IFN-*γ* secretion compared to PBMC for 3/8 subjects (2 infected subjects of 3 months and 12 years of age and one 14-month-old-vaccinated child). These results are consistent with those obtained by the addition of blocking anti-MHC class I antibodies and suggest that FHA-induced CD8^+^ lymphocytes can sometimes inhibit the FHA-induced CD4^+^ T-cell-mediated IFN-*γ* synthesis, whereas in other circumstances, CD8^+^ lymphocytes represent a significant source of IFN-*γ*.

## 4. Discussion

The comprehensive knowledge of the immune responses elicited by infectious agents that induce long-term protective immunity in infected patients may be instrumental for the design of optimal vaccine strategies against these microbes. Whereas most work on the adaptive cellular immune responses to extracellular pathogens has so far focused on the CD4^+^ T-cell responses, little is known on the possible induction of specific CD8^+^ T-cell responses. The susceptibility of infants to several severe infections with extracellular bacteria is partially attributed to a delayed maturation of their CD4^+^ T-cell responses with a delay in the IFN-*γ* secretion [4]. However, in addition to antigen-specific CD8^+^ T-cell responses in CMV- and *Trypanosoma cruzi*-infected infants [11, 12], the data reported here indicate that *B. pertussis* infection also can result in CD8^+^ T-cell-mediated IFN-*γ* responses, in addition to CD4^+^ responses. As IFN-*γ* is one of the factors that play a role in protection against *B. pertussis* infection [26], these data provide new insights in the mechanisms of protective immunity against *B. pertussis*, a major pathogen in view of the high prevalence of whooping cough worldwide in spite of extensive use of efficacious vaccines. The incidence of whooping cough is actually rising in countries with high vaccine coverage with a 5-fold increase in the number of whooping cough cases reported in June 2010 in California, compared to the same time period in 2009 [27].

The dual roles of CD4^+^ and CD8^+^ lymphocytes in the FHA-induced IFN-*γ* production were determined by the use of blocking anti-MHC class I or anti-MHC class II antibodies and by flow cytometry. This is in contrast to the classical concept that after phagocytosis of bacteria, their antigens are presented to CD4^+^ T lymphocytes by an MHC-class II-dependent pathway. Exceptions to this rule have been reported for intracellular bacteria, such as mycobacteria, which were shown to also induce CD8^+^ responses [28–30]. However, to our knowledge, the involvement of CD8^+^ T cells in the cellular immune responses to extracellular bacteria, such as *B. pertussis*, has not been addressed in details previously.

It has been shown that FHA can bind to CR3, which may result in uptake of the bacteria in CR3-expressing cells [31–33] and inhibition of the phagosome-lysosome fusion [31, 34]. FHA may then be presented by MHC class I via a cross-presentation pathway, as already demonstrated for intracellular bacteria [35, 36]. Alternatively, as FHA was shown to strongly bind to adenylate cyclase toxin (CyaA) [37], we can therefore not formally exclude a slight contamination of the FHA preparation by CyaA. This protein may then serve as a vector for cytosolic delivery of FHA, thereby allowing its presentation to CD8^+^ lymphocytes through the MHC class I molecules [38, 39]. CyaA has previously been shown to be directly delivered into the cytosolic pathway for MHC class I-restricted antigen presentation [40].

Even though both CD4^+^ and CD8^+^ lymphocyte subsets participate in the FHA-specific IFN-*γ* production, the major source of FHA-specific IFN-*γ* is the CD4^+^ T-lymphocyte subset in most of the infected or vaccinated children. Interestingly however, the CD4^+^ T-cell-mediated IFN-*γ* secretion appears to be modulated at least in some children by CD8^+^ lymphocytes, as shown by the enhanced FHA-induced IFN-*γ* concentrations measured in the cell culture supernatants after CD8^+^ T-cell depletion or in the presence of blocking anti-MHC-I antibodies. CD8^+^ lymphocytes have been reported in different human infections to secrete inhibitory cytokines, such as IL-10 or IL-4 [41–43], and CD8^+^CD25^+^ regulatory T cells have also been reported to suppress the CD4^+^ T-cell-mediated IFN-*γ* production and proliferation [44, 45]. Such cells may be induced to avoid excessive inflammatory responses during whooping cough or in response to pertussis vaccines.

On the other hand, the FHA-induced IFN-*γ* synthesis by CD8^+^ T lymphocytes was most often dependent on the CD4^+^ lymphocytes, as shown by the sharp decrease in IFN-*γ* secretion by CD8^+^ lymphocytes in the absence of CD4^+^ lymphocytes. The concept that helper CD4^+^ T cells may be essential for CD8^+^ T-cell primary response was established by different studies in recent years [46, 47], and several mechanisms have been proposed. Antigen-presenting cells activated by the CD4^+^ lymphocytes through CD40-CD40L interaction may stimulate the response of CD8^+^ T lymphocytes. For *B. pertussis *antigens, the antigen-presenting cells may be B lymphocytes expressing CD40 that were suggested to be able to present FHA to T lymphocytes [[23] and our unpublished data]. Alternatively, CD8^+^ T cells may be primed by the release of cytokines, such as IFN-*γ* and IL-2 by the CD4^+^ T lymphocytes, as was reported for the priming of cytotoxic effectors CD8^+^ cells [48].

The role of the FHA-induced CD8^+^ T-cell responses in protection is not known. No protective role of CD8^+^ T cells was demonstrated in *B. pertussis*-infected mice so far [49–51]. However, whole cell pertussis vaccine has been used in a mouse model as adjuvant to induce a cytotoxic activity against a tumor peptide [52]. It is therefore possible that the FHA-induced IFN-*γ* production by CD8^+^ lymphocytes is accompanied by a cytotoxic function of these cells. On the other hand, the CD8^+^ T-cell-mediated IFN-*γ* secretion may reflect the adjuvant activity of *B. pertussis*, and the role of *B. pertussis*-specific CD8^+^ lymphocytes may be different in humans and in mice. Whether a cytotoxic activity against *B. pertussis* may be helpful for protection against *B. pertussis *infection in humans or whether the antigen-specific CD8^+^ T cells mostly play a role in the control of the CD4^+^ response remains to be determined. However, the involvement of both the CD4^+^ and the CD8^+^ lymphocytes in the IFN-*γ* response induced by extracellular bacteria suggests a role of a crosstalk between these lymphocyte subpopulations in the regulation of the adaptive immune response to bacteria.

## 5. Conclusion

Although CD4^+^ T-cell responses to *B. pertussis* antigens have been extensively studied, little is known about CD8^+^ T-cell responses to these antigens in *B. pertussis*-infected or vaccinated children. In fact, CD8^+^ T-cell responses in bacterial infections or to nonlive vaccines have attracted limited attention so far, in contrast to viral or intracellular parasite infections. Here, we found that *B. pertussis* infection of infants with a median age of 2 months or young adults induces a strong IFN-*γ* response to FHA, a major *B. pertussis* antigen, and that the FHA-specific IFN-*γ* response is mediated by both CD4^+^ T cells and CD8^+^ T cells. Although the CD4^+^ T cells constitute the major source of FHA-specific IFN-*γ*, in some children substantial amounts of IFN-*γ* were produced by the CD8^+^ T cells. This was determined by the use of specific inhibitory anti-MHC class I and MHC-class II antibodies, as well as by flow cytometry, which showed that the major FHA-specific IFN-*γ*-secreting cell populations were the CD4^+^ T cells, both in infected and in vaccinated children. Cell depletion experiments showed that CD8^+^ T-cell-mediated IFN-*γ* secretion depends on the help of CD4^+^ T cells. Interestingly, in some children, depletion of the CD8^+^ T cells resulted in a substantial increase of FHA-specific IFN-*γ*, suggesting that these CD8^+^ T-cells may have immunoregulatory functions. Thus, a bacterial infection with *B. pertussis* or vaccination with nonlive vaccines, such as the whole-cell or the acellular pertussis vaccines, may induce a strong CD8^+^ T-cell response in children. The role of this response in protection against pertussis awaits further investigation.
